# Improving Radiation Resistance of GaInP/GaInAs/Ge Triple-Junction Solar Cells Using GaInP Back-Surface Field in the Middle Subcell

**DOI:** 10.3390/ma13081958

**Published:** 2020-04-22

**Authors:** Hui Gao, Ruixia Yang, Yonghui Zhang

**Affiliations:** 1 School of Electronics and Information Engineering, Hebei University of Technology, Tianjin 300401, China; 2 Tianjin Institute of Power Sources, Tianjin 300384, China; 3 The Key Laboratory of Electronic Materials and Devices of Tianjin, Tianjin 300401, China

**Keywords:** radiation resistance, electron beam irradiation, GaInP/GaInAs/Ge triple-junction space solar cell, back-surface field

## Abstract

This paper studies the radiation resistance for GaInP/GaInAs/Ge triple-junction space solar cells with a GaInP back-surface field (BSF) in the GaInAs middle subcell compared with those with an AlGaAs BSF. The results show that the initial electrical performance is almost the same for both of them. However, the radiation resistance of the GaInP BSF cell was improved. After irradiation by 1 MeV electron beam with a cumulative dose of 10^15^ e/cm^2^, the *J_sc_* declined by 4.73% and 6.61% for the GaInP BSF cell and the AlGaAs BSF cell, respectively; the efficiency degradation was 13.64% and 14.61% for the GaInP BSF cell and the AlGaAs BSF cell, respectively, leading to a reduced degradation level of 6%. The mechanism for GaInP BSF to improve the radiation resistance of GaInP/GaInAs/Ge triple-junction solar cells is also discussed in this work. Similar results were obtained when irradiation cumulative doses varied from 1 × 10^14^ e/cm^2^ to 1 × 10^16^ e/cm^2^.

## 1. Introduction

GaInP/GaInAs/Ge lattice-matched triple-junction solar cells have been widely used in space photovoltaic applications and have attained the highest efficiency over 30% [1,2]. The heavy radiation bombardment with various energetic particles in a space environment will inevitably damage the solar cells and result in additional non-radiative recombination centers, which reduces the minority carrier diffusion length and leads to degradation of the solar cell efficiency [3]. The subcells in multi-junction solar cells are connected in a series; the subcell with the highest radiation degradation impairs the efficiency of the multi-junction solar cells. It has been established that GaInAs subcells restrict the radiation resistance of GaInP/GaInAs/Ge solar cells, because there is a higher migration energy of V_Ga_ (1.79 eV) and V_As_ (1.48 eV) in GaAs compared with that of V_In_ (0.26 eV) and V_P_ (1.2 eV) in InP [4,5]. Measures such as reducing the doping concentration, thinning the thickness of the base region, and so on can be taken to improve the radiation resistance of the GaInAs subcells [6,7,8,9].

The back-surface field (BSF) has a great influence on the performance of solar cells. To enable more carriers to arrive at the depletion region, a sufficient conduction band discontinuity can be used as an effective reflector for minority carriers [10]. The BSF material needs to be lattice matched with the base material, otherwise a high dislocation density will be caused by the mismatch and can lead to the rapid recombination of minority carriers, which will seriously affect the photovoltaic efficiency of the solar cell [11]. GaInP/GaInAs/Ge triple-junction solar cells containing BSFs in GaInAs middle cells with materials of GaInP, AlGaAs, etc. are being widely studied at present [12,13]. Ga_0.5_In_0.5_P BSF behaves as an outstanding minority carrier reflection but includes a non-negligible increase in the series resistance of the device. AlGaAs BSF exhibits superior performance both in terms of mirror for minority carriers and series resistance [14]. However, the role of BSF materials in the radiation resistance of solar cells has not been studied so far. 

In this work, we fabricated GaInP/GaInAs/Ge triple-junction solar cells, applying a GaInP layer or an AlGaAs layer as the BSFs of GaInAs middle subcells, respectively. The performances for both types of cells were measured and compared before and after energetic electron irradiation. We found that substituting the BSF makes no difference to the initial characteristics of the GaInP/GaInAs/Ge triple-junction solar cell. However, the radiation resistance of a triple-junction solar cell with a GaInP BSF is much better than that of one with an AlGaAs BSF. The mechanism for the GaInP BSF to improve the radiation resistance of a GaInP/GaInAs/Ge triple-junction solar cell was interpreted based on both the high electric fields of the GaInAs base region/GaInP BSF layer heterostructure and the reduction of the interface state density.

## 2. Experiment 

**Figure 1:**
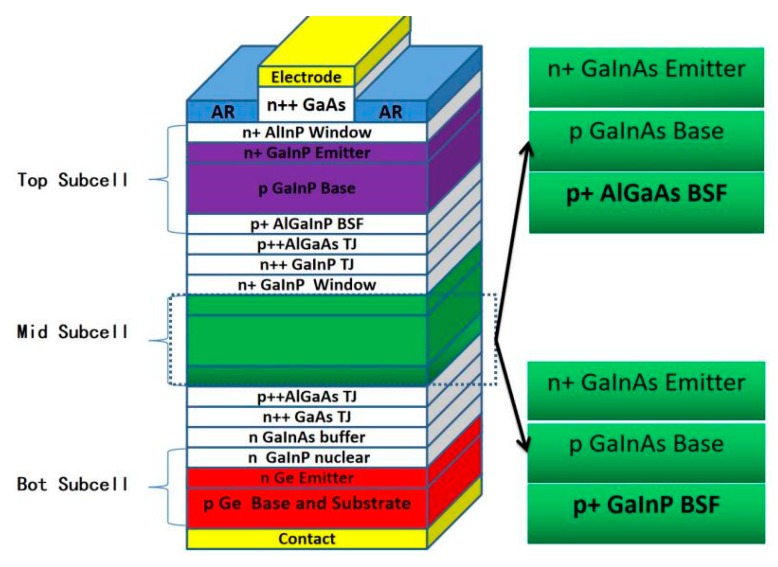
Schematic structure for solar cells with an AlGaAs back-surface field (BSF) or a GaInP BSF in the middle subcell, respectively.

All of the epitaxial layers were grown in a commercial planetary metal organic chemical vapor deposition reactor (MOCVD) (Aixtron 2800, Aachen, Germany) at the growth pressure of 50 mbar. Four-inch p-type-doped (001) Epi-ready Ge wafers (175 μm) with 9° miscut toward the (111) plane were used as growth substrates. Hydrogen (H_2_) was used as the carrier gas. Trimethylgallium (TMGa), trimethylindium (TMIn), trimethylaluminum (TMAl), arsine (AsH_3_), and phosphine (PH_3_) were used as precursors for Ga, In, Al, As, and P, respectively. Diethylzinc (DEZn) and silane (SiH_4_) were utilized as p-type and n-type dopants, respectively. A more detailed preparation process and process parameters can be observed in the literature [15]. Two types of solar-cell structures used in this work are shown in Figure 1. The two cell structures are identical except the BSF layer. The thickness was 30 nm, and the doping concentration was 2 × 10^18^ cm^−3^ for both the GaInP BSF and the AlGaAs BSF layers. The details for the BSF material growth are as follows: The Al_0.2_Ga_0.8_As BSF layer was grown at a temperature of 680 °C, with a growth rate of 6 µm/h, and a V/III ratio of 102. The Ga_0.505_In_0.495_P BSF layer was grown at a temperature of 660 °C, a growth rate of 1.4 µm/h, and a V/III ratio of 300. In order to avoid a lattice mismatch with the GaInAs subcell caused by a GaInP composition deviation, part of the functional layer of the solar cell from the Ge substrate to the BSF was prepared first, with the thickness of the BSF increased to 1.3 μm. The omega-2theta diffraction curve was measured for the lattice constant of the BSF layer by a high-resolution four-crystal diffractometer (PANalytical MRD, Eindhoven, The Netherlands), which included a Cu X-ray generator and a pixel 30 detector. The test results were peak fitted with the simulation results using their own calculation software. After that, the entire solar wafers were fabricated.

Solar wafers were processed into devices by applying the same processing techniques. The ohmic contacts on the front and back electrodes were made, i.e., finger electrodes of Ge/Au/Ag film were deposited on the front surface using electron beam evaporation and photolithographic processing. Ag/Au film was then deposited on the reverse side and subsequently annealed at the temperature of 400 °C for 5 min in N_2_ atmosphere. Isolated etching and sawed dicing were used to produce bare solar cells with a chip size of 6 × 4 cm^2^. Finally, TiO_2_/Al_2_O_3_ anti-reflective coating (ARC) was deposited onto the front surface of the bare solar cells by an electron beam (e-beam) evaporator.

The solar cell samples were then prepared for e-beam irradiation experiments by an ELV-8II electron accelerator with a cumulative dose of 10^15^ e/cm^2^ and an energy of 1 MeV. The temperature of the accelerator chamber was lower than 50 °C. After the irradiation, an annealing treatment was done at 28 °C/AM0 for 48 h. Before and after the irradiation experiment, the performance parameters of the solar cells were evaluated. The light I-V characteristics were measured under AM0 illumination condition (1365 W/m^2^, 25 °C) using a SourceMeter (Keithley 2602B, Beaverton, OR, USA) and a solar simulator (Spectrolab X25, Sylmar, LA, USA). To discover the irradiation-induced changes of the subcells, a spectral external quantum efficiency (EQE) was performed by an alternative current type (Enlitech QE-R3018, Kaohsiung, Taiwan) across a wavelength range of 300–1800 nm. In order to characterize the degree of attenuation at different doses, radiation attenuation experiments with different radiation doses from 10^14^ e/cm^2^ to 10^16^ e/cm^2^ were also performed.

## 3. Results and Discussion

### 3.1. X-ray Diffraction Characterization of the GaxIn_1−x_P BSF Layer

Dislocations and poor surface roughness can affect the experimental results. AlGaAs BSF homogeneity epitaxy is different from that of a GaInP BSF, which has to be heteroepitaxy grown following AlGaAs tunnel junction. According to the Vegard Law [16], the lattice constant of GaInP can be expressed as Formula (1):

(1) $$a_{Ga_{x}In_{1-x}P}=5.8687-0.4182x$$

where *x* is the composition of Ga in the material.

**Figure 2:**
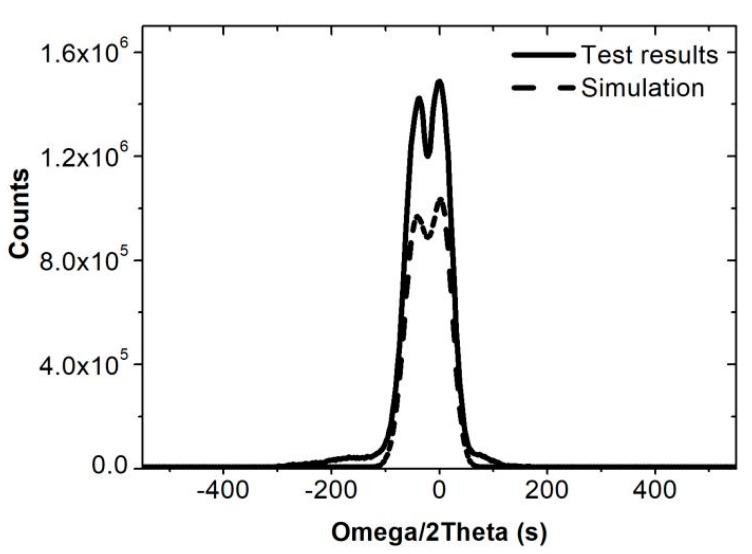
Omega-2Theta curves of the GaInP BSF layer and simulated curves.

A designed *x* value of 0.505 for the *Ga_x_In*_1−*x*_*P* layer was chosen to make the *Ga_x_In*_1−*x*_*P* BSF lattice match with the Ge substrate. We grew the structure from the Ge substrate to the GaInP BSF and intentionally increased the thickness of the GaInP BSF to 1.3 μm in order to test the lattice parameters. The high-resolution X-ray diffraction (HRXRD) measurements were carried out by a Panalytical X’Pert MRD device. The omega-2theta diffraction curve is shown in Figure 2. The solid line is the omega-2theta diffraction curve of (004) reflection. Only two peaks can be identified, mainly because other materials either overlapped the Ge peak or were too thin. The design layers can be simulated by the instrument’s own simulation software to fit the results. Considering that the BSF layer was far thicker than other layers, we directly simulated a 175 μm Ge material with 1.3 μm Ga_0.502_In_0.498_P deposited, and the material simulation curves (the dashed line) are also shown in Figure 2. It can observed that the two peak locations for the tested curve and simulated curve are almost perfectly consistent. Therefore, we can verify that the GaInP BSF material we grew is Ga_0.502_In_0.498_P, which is approaching the designed composition of Ga_0.505_In_0.495_P. In addition, we can find from the figure that the full width at half maximum is only about 55 arcsec, a small value for the tested sample, which means that the crystal quality is excellent.

### 3.2. Geosynchronous Orbit Anti-Irradiation Performance

**Figure 3:**
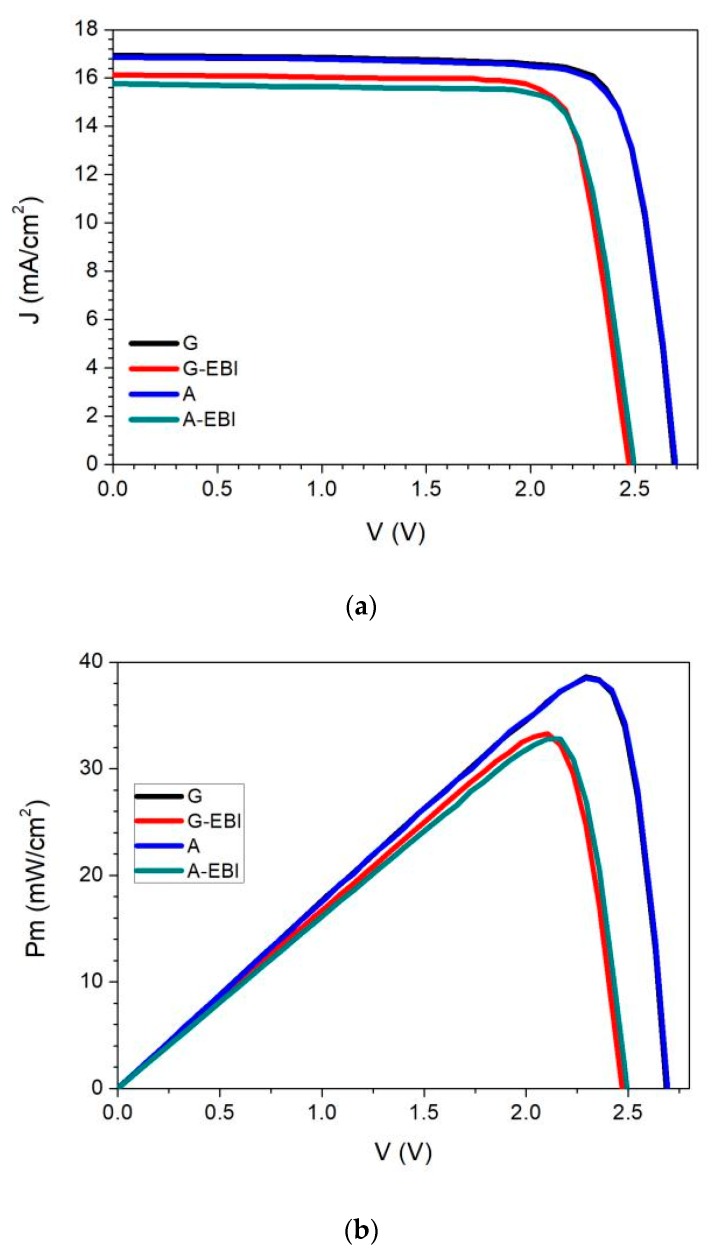
Light I-V characteristics before (G, A) and after electron beam irradiation (G-EBI, A-EBI): (**a**) Current density vs. voltage curve; (**b**) Power density vs. voltage curve.

The light I-V characteristics and EQE for the two types of solar cells were measured before and after electron beam irradiation. For convenience, the cells with GaInP BSF are denoted as Device G, while those with AlGaAs BSF are denoted as Device A. After Device A and Device G were processed by electron beam irradiation, they were named Device A-EBI and Device G-EBI, respectively. The measurement results for all samples are shown in Figure 3. The values of short circuit current density (*J_sc_*), open circuit voltaic (*V_oc_*), and max power (*P_m_*) for the G samples and A samples before and after irradiation are given in Table 1. Table 1 shows the average and standard deviation of the test results of four samples for each type.

As can be seen from Figure 3, the light I-V characteristics for Device A and Device G were almost the same, which indicates that the initial performance of solar cells before electron beam irradiation was little affected by the two types of BSF. All values decreased as a result of the electron irradiation, and the *J_sc_* attenuation for G-EBI was low compared with that for A-EBI in Figure 3a. The change trend of *V_oc_* is also shown in Figure 3a. The *V_oc_* values decreased for all the samples owing to electron irradiation-induced damage. However, the *V_oc_* attenuation for the G samples was higher than that for the A samples. Figure 3b indicates the *P_m_* before and after electron irradiation. Sample G and Sample A had almost the same *P_m_* value before electron irradiation. Irradiation caused the *P_m_* value to decrease for all samples, and the *P_m_* value for G-EBI was higher than that of samples A-EBI. The attenuation of voltage and current was the main manifestation of the performance attenuation.

As shown in Table 1, the initial average light I-V characteristics for the two types of cells were very close to each other. This implies that the alteration of the BSF does not significantly affect the initial performances for the solar cells. After the electron irradiation, the output parameters of the solar cells were obviously degraded. G-EBI exhibited better *J_sc_*, FF, and *P_m_* compared with A-EBI. The *J_sc_* decay rate for G samples was 4.7%, which was 30% lower than the value of 6.6% for the A samples. The efficiency degradation ratio for the G samples was 13.6%, while it reached 14.5% for the A samples, indicating that the former was 6% lower than the latter. It should be noted that the V decay rate of the G samples was higher than A, which may have been due to the high series resistance in the GaInAs mid subcells produced by the higher valence band offset for the GaInP/GaAs interface.

We define the index of anti-irradiation resistance characteristics with Formula (2):

(2) $$I_{r}=\left[\frac{J_{rad,sc}}{J_{0,sc}}\right]\times \left[\frac{V_{rad,oc}}{V_{0,oc}}\right]\times \left[\frac{P_{rad,m}}{P_{0,m}}\right]$$

where *I_r_* is the anti-irradiation resistance coefficient, *J*_0,*sc*_ is the short-circuit current density of the beginning of life (BOL), *J_rad,sc_* is the short-circuit current density of the end of life (EOL) after irradiation, *V*_0,*oc*_ is the open circuit voltage of BOL, *V_rad,oc_* is the open circuit voltage of EOL, *P*_0,*m*_ is the maximum power of BOL, and *P_rad,m_* is the maximum power of EOL.

This formula can characterize the decay characteristics of the voltage, current, and maximum power and comprehensively and quantitatively evaluate the irradiation decay characteristics of the solar cells. It can be calculated from this formula that the results of current part, voltage part, maximum power part, and the *I_r_* are 0.953, 0.916, 0.864, and 0.7542 for G and 0.934, 0.926, 0.855, and 0.7395 for A. G had better radiation resistance parameters than A. The larger contribution of the current part was the key factor.

**Figure 4:**
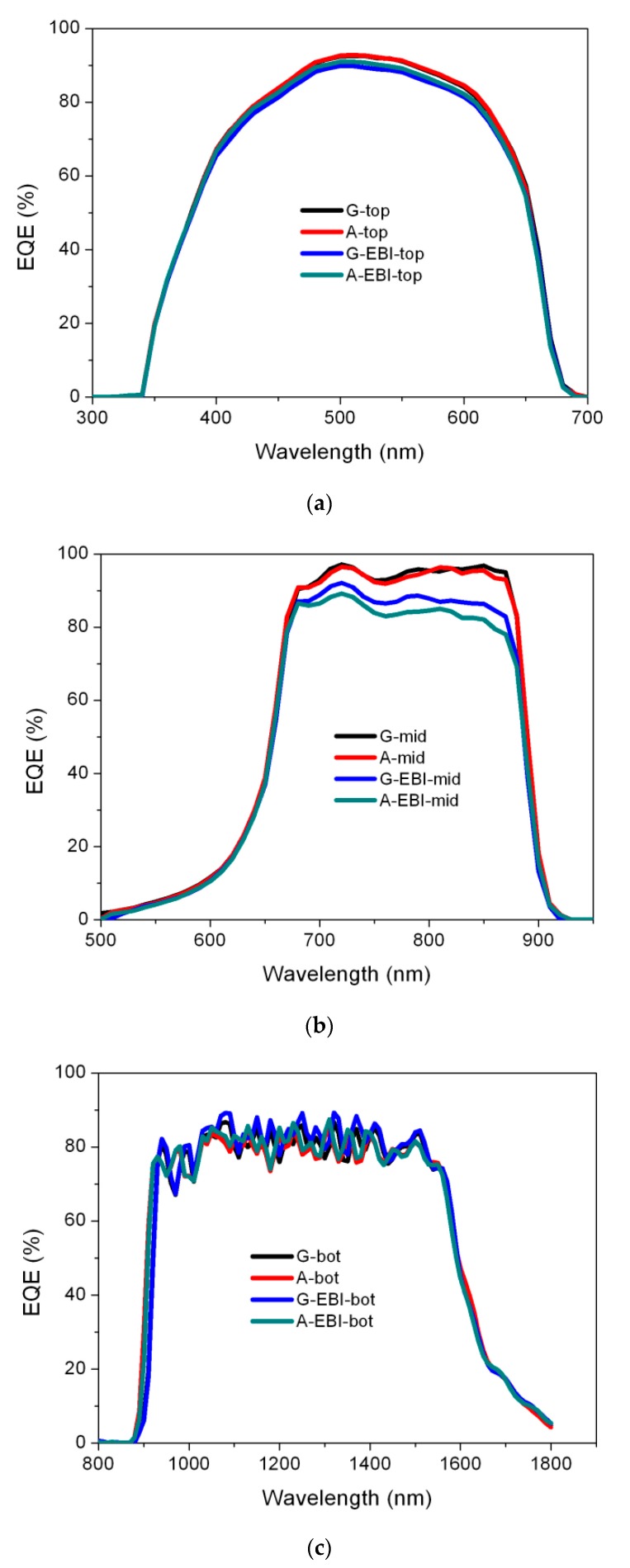
External quantum efficiency (EQE) curves of solar cells before (G, A) and after (G-EBI, A-EBI) electron beam irradiation: (**a**) EQE result of top subcells; (**b**) EQE result of middle subcells; (**c**) EQE result of bottom subcells.

The degradation of the output power results from a change in the subcell operating points. In order to identify which subcell had the most degradation under the irradiation, we investigated the EQE measurement for each subcell individually before and after the electron beam irradiation shown in Figure 4. 

The corresponding subcells for Sample G and Sample A had the same response range for the wavelength, and their EQE at the corresponding wavelength almost coincided with each other, so the current densities of subcells were nearly the same. For Device G, the current density levels for the subcells that were estimated from the integration of the EQE spectrum with the AM0 spectrum were 17.64 mA/cm^2^, 17.794 mA/cm^2^, and 26.96 mA/cm^2^ from top to bottom, respectively. For A, the corresponding results were 17.61 mA/cm^2^, 17.763 mA/cm^2^, and 26.587 mA/cm^2^, respectively. 

As shown in Figure 4, the EQE results for the two samples were obviously different after irradiation. We found that the response range for the wavelength of their top subcells were almost the same, and the values were also highly consistent. The response range for the wavelength of their middle subcells were the same, but the EQE results appeared to be different. Sample G-EBI had a higher EQE value than Sample A; the value was improved significantly in the ranges from 750 to 850 nm. The current densities for the subcells, from top to bottom, were 17.217 mA/cm^2^,16.45 mA/cm^2^, and 26.909 mA/cm^2^, respectively. For Sample A-EBI, the corresponding results were 17.257 mA/cm^2^, 16.029 mA/cm^2^, and 26.781 mA/cm^2^, respectively. The results indicate a better radiation resistance of Sample G, which benefited from the improvement of the radiation resistance of the middle subcell. The EQE of its middle subcell was significantly higher than that of A after irradiation, especially in the long-wavelength range, which benefitted from the use of the GaInP back-surface field structure.

The performance degradation caused by radiation for multi-junction solar cells was mainly due to the generation of recombination centers. Our experimental results indicated that the middle subcell with a GaInP BSF exhibited a better anti-radiation capability than the middle subcell with an AlGaAs BSF. We believe that the mechanism contains two aspects: one is related to the difference between the conduction band offsets for the base region/BSF hetero-junctions of the two types of middle subcells, and the other is related to the interface states of the base region/BSF layer interface.

In the middle subcell structure, the doping concentrations in the BSF layer were higher than those in the base region, and the p-type BSF layer can be treated as a p^+^ layer. Thus, a p^−^p^+^ junction is built by a p-type base region and p^+^ BSF layer. The electric field as well as the potential barrier for electrons was generated at the p^−^p^+^ junction. The incoming electrons will encounter a reflecting effect by the electric field in the p-type base region, and the potential barrier will push them back to the p-n junction depletion region of the middle subcell. Then, with the help of a built-in electric field in depletion region, these electrons will drift to the n-type emitting region, which is helpful to improve the transport capability of the carrier far from the p-n junction depletion zone [17,18].

**Figure 5:**
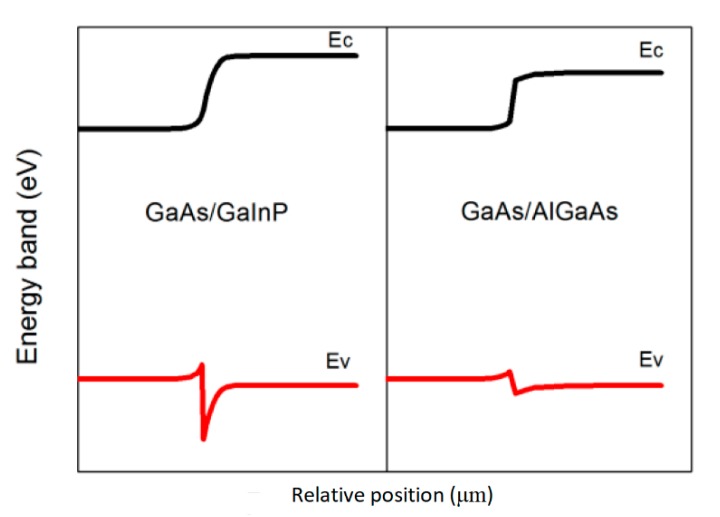
Band diagram of a p-GaAs/pAl_0.2_Ga_0.8_As heterojunction. (Right) band diagram of a p-GaAs/pGa_0.5_In_0.5_P heterojunction. In both cases the doping level is constant throughout the heterojunction and equals N_A_ = 2 × 10^18^ cm^−3^.

Before irradiation, the electrical properties for the two types of solar cells were almost the same. However, the EQE curve indicates that the value of the intermediate cell using GaInP BSF was slightly higher than that of the subcell with AlGaAs BSF in the 800–900 nm spectral range. We believe that this can be ascribed to the wider band gap of GaInP (1.87 eV) compared to that of AlGaAs (1.67 eV). As shown in Figure 5, the wider band gap of GaInP leads to the result that the conduction band offset of the GaInAs/GaInP heterostructure is greater than that of the GaInAs/AlGaAs heterostructure, and therefore, a larger electric field is built at the GaInAs/GaInP heterostructure. Thus, the reflecting effect of the GaInP BSF was stronger compared to that of the AlGaAs BSF. In other words, a GaInP BSF can more efficiently push electrons coming from the base region back to p-n junction depletion region of the middle subcell.

After irradiation, the minority carrier lifetimes decrease in the base regions of the two types of middle subcells because of the radiation-induced recombination centers, so the number of minority carriers that can reach the depletion zone will decrease. However, the degree of decrease is weaker for the subcell with a GaInP BSF than those with an AlGaAs BSF. This is because the strong electric field in the GaInAs/GaInP heterostructure can make minority carriers in the base region drift towards the p-n junction depletion region with a drift velocity greater than that for the GaInAs/AlGaAs heterostructure. The great minority carrier drift velocity in the subcell with a GaInP BSF, as compared to that in the subcell with an AlGaAs BSF, can more efficiently compensate for the effect of the minority carrier lifetime decrease on *J_sc_* of the middle subcell. Furthermore, due to the high radiation resistance of the GaInP crystal, the radiation-induced defects on the GaInAs/GaInP interface are fewer than those on the GaInAs/AlGaAs interface [19]. The decrease of the interface defects diminishes the interface recombination probability and is also helpful for the increase of *J_sc_*. Thus, we can conclude that a GaInP BSF, as compared to the traditional AlGaAs BSF, can provide a stronger drift electric field for minority carriers and can also diminish the radiation-induced defects of the base region/BSF layer interface. This is the main mechanism for the improved radiation resistance of the GaInAs middle subcell with a GaInP BSF.

### 3.3. The Degradation Trends of I-V Characteristics under Different Irradiation Doses

**Figure 6:**
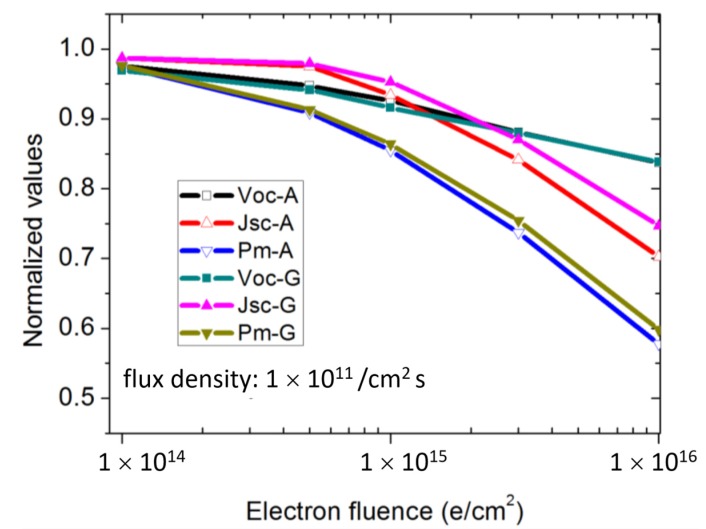
*J_sc_*, *V_oc_*, and *P_max_* values of Sample A and G solar cells irradiated by 1 MeV electron beam with flux density of 1 × 10^11^ e/cm^2^.

At present, the widely used space cells generally apply the radiation dose of the geosynchronous orbit for radiation resistance research, but radiation attenuation experiments with different radiation doses are also necessary to verify the radiation resistance of such cells. Figure 6 shows the degradation trends of *J_sc_*, *V_oc_*, and *P_m_* of the two different types of solar cells against the electron irradiation fluence with flux densities of 1 × 10^11^ e/cm^2^. All *J_sc_*, *V_oc_*, and *P_m_* values decreased with the increase of irradiated electron fluence amount in both cases. Comparing the effect of different structure design, the *V_oc_* degradation of Sample G was slightly larger than that of Sample A under all electron fluence amounts, while the *J_sc_* attenuation of Sample A was greater than those of Sample G, resulting in the bad *P_m_* attenuation of Sample A. These results fit well with the geosynchronous orbit anti-irradiation performance.

As has been mentioned previously, the middle subcell (especially its current parameters) limits the performance of the GaInP/GaInAs/Ge triple-junction solar cell, so the improvement of the middle subcell current parameters can increase the total performance of GaInP/GaInAs/Ge triple-junction solar cells. We suggest that a GaInP BSF is a good choice for middle subcell structure design of GaInP/GaInAs/Ge triple-junction solar cells for space application.

## 4. Conclusions

The relationship between BSF and anti-irradiation capabilities of the middle subcell in GaInP/GaInAs/Ge triple-junction solar cell was studied. GaInP and AlGaAs materials were applied as the BSFs of middle cells, respectively. The experimental results show that the GaInP BSF design did not show significant influence on the initial electrical performance of the solar cell device compared with the AlGaAs BSF. However, it effectively improved the radiation resistance under geosynchronous orbit anti-irradiation performance. The *J_sc_* declined 4.73%, which was reduced by nearly 30% compared with the solar cell using an AlGaAs BSF (6.61%). The degradation of *V_oc_* had a value of 8.4%, increased by 14% more than the solar cell applying the AlGaAs BSF (7.4%). The final efficiency decay rate was 13.64%, which decreased by 6% compared with the one using an AlGaAs BSF (14.51%). Similar results were obtained under more irradiation cumulative doses from 1 × 10^14^ e/cm^2^ to 1 × 10^16^ e/cm^2^.

## Figures and Tables

**Table 1:** Comparison of the I-V characteristics of the two types of solar cells before and after electron beam irradiation.

	Using GaInP BSF				Using AlGaAs BSF			
	*J_sc_* mA/cm^2^	*V_oc_* V	FF	*P_m_* mW/cm^2^	*J_sc_* mA/cm^2^	*V_oc_* V	FF	*P_m_* mW/cm^2^
Before irradiation	16.930 ± 0.017	2.6956 ± 0.001	0.8464 ± 0.0002	38.628 ± 0.0406	16.863 ± 0.013	2.6898 ± 0.002	0.8491 ± 0.0003	38.506 ± 0.1759
After irradiation	16.135 ± 0.021	2.4710 ± 0.0016	0.8375 ± 0.0018	33.311 ± 0.0135	15.762 ± 0.021	2.4960 ± 0.0028	0.8339 ± 0.0013	32.810 ± 0.0338
Degradation ratio (%)	4.7	8.4	1	13.6	6.6	7.4	1.1	14.5
